# Chronic Urticaria Suspected to be Caused by a 13.5 mg Levonorgestrel Intrauterine Device (Skyla®)

**DOI:** 10.7759/cureus.42287

**Published:** 2023-07-22

**Authors:** Malika P Ganguli, Vesselin Dimov

**Affiliations:** 1 Internal Medicine, Ross University School of Medicine, Bridgetown, BRB; 2 Allergy and Immunology, Cleveland Clinic Hospital of Florida, Weston, USA

**Keywords:** immunology, autoimmune progesterone dermatitis, iud, levonorgestrel, allergy, skyla, progesterone, intrauterine device, rash, chronic urticaria

## Abstract

Chronic urticaria (CU) is a hive-like rash lasting over six weeks. Common associations include low vitamin D, thyroid autoantibodies, and *Helicobacter pylori* (*H*. *pylori*) infection, among others. Progesterone has been documented to trigger CU, by endogenous or exogenous progesterone. The use of intrauterine devices (IUDs) has been a popular source of birth control, with many containing progesterone. Although rarely reported, some patients have been seen to have an urticarial reaction after implantation of an IUD. Here, we present a case of a patient with progesterone-induced chronic urticaria, likely triggered by implantation of a 13.5 mg intrauterine device implant (Skyla®, Bayer, Whippany, NJ, USA). To the best of our knowledge, this is the first case to report the association between Skyla® and chronic urticaria.

## Introduction

Chronic urticaria (CU) is a hive-like rash lasting over six weeks and a frustrating diagnosis for patients as it causes uncomfortable and potentially life-threatening symptoms such as severe pruritus and angioedema [1]. Although some common associations include low vitamin D and *Helicobacter pylori* (*H. pylori*) infection, the presence of autoimmunity is a known association with chronic urticaria such as thyroid autoantibodies and antinuclear antibodies with a speckle pattern [2]. Interestingly, autoantibodies produced in myasthenia gravis (anti-acetylcholine receptor) and type 1 diabetes (insulin receptor) have also been reported to have an association with CU [2]. Urticarial lesions may also occur in the event of heat or cold exposure, sunlight, and mechanical pressure on the skin [1]. Sex hormones, particularly progesterone, are rarely documented to trigger CU [2,3]. Progesterone is endogenously produced in the human body. In females, the production of progesterone varies due to the cyclical pattern of ovulation and menstruation. Females with progesterone hypersensitivity or “autoimmune progesterone dermatitis” may have recurrent monthly outbreaks of urticarial lesions [1-3]. Some females may be sensitized to progesterone; however, they may not have cyclical urticaria and may only show symptoms with the addition of exogenous progesterone, such as when taking birth control [3-5]. In this report, we explore a chronic urticarial rash in a young female, which seems to be associated with her 13.5 mg levonorgestrel (progesterone)-releasing intrauterine device.

## Case presentation

A 38-year-old patient presented to the clinic with complaints of a chronic rash for two years and uncontrolled chronic and allergic rhinitis. Past medical history was positive for intermittent asthma, migraine, polycystic ovaries, and pseudotumor cerebri. Past surgical history was positive for lithotripsy of a kidney stone, cholecystectomy, and meniscal repair of the knee. Social history was negative for tobacco and drug use and positive for social alcohol use. Current medications include cetirizine, diphenhydramine, and Skyla® intrauterine device (IUD) (Bayer, Whippany, NJ, USA). Her cutaneous lesions were similar to those in chronic urticaria (CU), with wheals and flares on her arms, torso, and legs. The patient denied pruritus or systemic symptoms. Suspected triggers for the rash were cinnamon, hair products, and over-the-counter coenzyme Q10. The lesions continued after stopping the coenzyme supplement.

Allergen patch tests were negative for cinnamon but positive for fragrance and iodopropynyl butylcarbamate (common additives in cosmetic products), and she was advised to avoid contact with these substances. A blood test suggested a class 1 hypersensitivity to cat dander (IgE 0.51 kU/L) and a class 2 hypersensitivity to short ragweed (IgE 1.14 kU/L). Furthermore, skin prick allergy tests were positive for house dust mites, weed pollen, neomycin sulfate, chicken, cod, fish, lettuce, and mushroom. She was advised to avoid all allergens. An extensive workup for CU was done. Anti-thyroglobulin antibodies, hepatitis C, and *Helicobacter pylori* carrier status were negative. Antinuclear antibodies, C1 esterase inhibitor, C3, C4, complement deficiency assay, C-reactive protein, serum IgE, microsomal antibody, rheumatoid factor, thyroid-stimulating hormone, and serum tryptase were within normal limits. Urticaria-inducing activity was 0, suggesting no influence of basophil antibodies. Serum IgE levels were within normal limits at 51 kU/L. Pregnancy test was negative. Venereal disease research laboratory (VRDL) test was non-reactive. She was borderline vitamin D deficient at 29.5 ng/mL. The patient had urticarial lesions two years prior to vitamin D testing and was treated with oral supplementation. Nine months later, her vitamin D levels were found to be corrected at 55 ng/mL; however, she continued to have CU lesions.

She was treated with montelukast and triamcinolone nasal spray for chronic and allergic rhinitis and albuterol for asthma, which provided adequate control of symptoms. For her chronic urticaria, she was prescribed an epinephrine auto-injector in the event she has acute angioedema with airway restriction, along with daily fexofenadine and avoidance of allergens. She continued to have urticarial lesions, although she was compliant with her new restrictions. At this time, we considered her 13.5 mg levonorgestrel intrauterine device (Skyla®) as a potential cause of CU, as the symptoms originally started five months post-implantation of her IUD. Progesterone (endogenous and exogenous) has a documented association with chronic urticaria. We advised implant removal, and it was removed by her gynecologist. The patient denied progesterone skin prick confirmatory testing as it is not standardized, in addition to the additional cost of the test, and the result will not change her course of clinical management. The patient was relieved to see the rash improving gradually, with complete resolution of CU symptoms two years post-IUD removal. We are unsure of her birth control usage history aside from the intrauterine device. The timeline of events is clarified in Table 1.

**Table 1:** Timeline of events

Year	Reason for visit
2011	First seen in the allergy clinic for rhinoconjunctivitis
2014	Intrauterine device implanted
2015	Seen in the allergy clinic for the emergence of chronic urticarial lesions and subsequently underwent blood, skin prick, and patch testing
Late 2015	Vitamin D deficiency resolved
2016	Intrauterine device removed with a slight resolution of urticarial lesions
2018	Complete resolution of chronic urticaria

## Discussion

Our best hypothesis for the cause of this patient’s chronic urticaria is due to her 13.5 mg intrauterine device (IUD), Skyla® (Bayer, Whippany, NJ, USA), due to the temporal relationship between the insertion of the device and the onset of rash along with the removal of the device and the improvement of rash. We cannot, however, confirm with absolute certainty that the IUD was the cause as there was no confirmatory skin prick test done on progesterone or any of the ingredients in Skyla® to identify a hypersensitivity reaction. It is possible that this patient had an episode of idiopathic chronic urticaria. However, we cannot ignore the temporal relationship between the onset and cessation of the rash and the IUD implantation and removal. Through otherwise extensive testing, we have also helped eliminate many possible contributors that may have an association with chronic urticaria, and our suspicion of the role of progesterone in the pathogenesis of this patient’s natural history of disease is a diagnosis of exclusion.

Medical literature on this phenomenon is severely limited. We searched the PubMed database for articles in English reporting the occurrence of urticaria with hormone exposure. One case reports the onset of solar urticaria related to the use of oral contraceptives, suggesting a hormonal link [6]. One case reports chronic urticaria in a patient after mifepristone exposure for a medically induced pregnancy termination [4].

The literature on chronic urticaria with IUDs is even further limited. IUDs are T-shaped contraceptive devices placed within the uterine cavity. There are several IUD options: a non-hormonal copper IUD, 52 mg levonorgestrel IUD (Mirena®, Bayer, Whippany, NJ, USA, and Lileta®, Allergan, Irvine and San Francisco, CA, USA), 19.5 mg levonorgestrel IUD (Kyleena®, Bayer, Whippany, NJ, USA), and 13.5 mg levonorgestrel IUD (Skyla®, Bayer, Whippany, NJ, USA). Non-hormonal components of Skyla® are made with polyethylene plastic, polydimethylsiloxane, colloidal silica, silver, barium sulfate, and iron oxide, similar to other IUDs. It contains 13.5 mg of the active ingredient levonorgestrel (progesterone) dispersed in a steroid reservoir and released at a rate of 14 mcg/day. Few papers reported chronic urticaria in copper-based IUDs; however, they were older than 20 years and not accessible in modern databases [7,8]. One case series reports chronic urticaria in seven patients with a levonorgestrel IUD. However, the study did not specify the dosages of progesterone in their IUDs. Thus, we cannot confirm if Skyla® caused symptoms in any of these patients [5]. Two papers described the incidence of acute urticarial rash in patients two hours after implantation of Mirena®, with a resolution of the rash after IUD removal [1,9]. Interestingly, one of these patients was already premedicated with an oral antihistamine for chronic allergic rhinitis prior to implantation and, regardless, had an acute urticarial rash [1]. One case reports a pruritic eruption on the chest, legs, and arms of a female after Mirena® was inserted [3].

Pathogenesis is poorly understood. There may be a combination of both type I and IV immune reactions through a T-helper (Th2) lymphocyte-mediated reaction [1,4,5]. Sensitization may occur from cross-sensitivity between progesterone products in the production of immunoglobulin E synthesis [4]. Sources for sensitization could be hormone contraceptives, emergency contraception, and mifepristone in the event of medically induced termination of pregnancy [4]. Further inflammation may result from the direct effect of progesterone on basophil and mast cell degranulation [4]. Testing is limited. Skin prick tests with levonorgestrel can be used to confirm progesterone hypersensitivity. However, the sensitivity and specificity of this test are not well documented, suggesting the need to develop a standardized vial of progesterone for testing [5]. Testing for CD203, a nonspecific marker for mast and basophil cell activation, has also been reported in the literature [5,10].

Management is tricky. Patients with endogenous progesterone sensitivity may require the use of antihistamines, corticosteroids, hormone antagonists, and, in severe cases, bilateral oophorectomy [1]. In exogenous sensitivity, removal of the offending agent and use of antihistamines and corticosteroids may be used. Long-term treatment may be achieved with oral or intramuscular immunotherapy for desensitization [1].

## Conclusions

In summary, we describe the case of a 38-year-old female with a chronic urticarial rash. The patient was compliant with the avoidance of allergens and in the resolution of vitamin D deficiency but continued to have urticarial lesions. We deduced that the trigger was due to exogenous progesterone secondary to her intrauterine device, Skyla®. Although rare, progesterone is a known cause of urticarial lesions. To the best of our knowledge, this is the first case to report the incidence of chronic urticaria in relation to the intrauterine device Skyla®.
